# Improved Bat Algorithm Based on Multipopulation Strategy of Island Model for Solving Global Function Optimization Problem

**DOI:** 10.1155/2019/6068743

**Published:** 2019-08-18

**Authors:** Sha-Sha Guo, Jie-Sheng Wang, Xiao-Xu Ma

**Affiliations:** ^1^School of Electronic and Information Engineering, University of Science & Technology Liaoning, Anshan 114044, China; ^2^School of International Finance and Banking, University of Science & Technology Liaoning, Anshan 114044, China

## Abstract

The bat algorithm (BA) is a heuristic algorithm that globally optimizes by simulating the bat echolocation behavior. In order to improve the search performance and further improve the convergence speed and optimization precision of the bat algorithm, an improved algorithm based on chaotic map is introduced, and the improved bat algorithm of Levy flight search strategy and contraction factor is proposed. The optimal chaotic map operator is selected based on the simulation experiments results. Then, a multipopulation parallel bat algorithm based on the island model is proposed. Finally, the typical test functions are used to carry out the simulation experiments. The simulation results show that the proposed improved algorithm can effectively improve the convergence speed and optimization accuracy.

## 1. Introduction

Optimization is the selection of the best elements for a particular set of criteria from a range of effective choices, which shows many different advantages and disadvantages in terms of computational efficiency and global optimization probability, but has a wide range of applications in industry and scientific research [1]. Function optimization proposes a formal framework for modeling and solving a series of specific problems, giving an “objective” function that takes a parameter as input, and the goal is to find the value of the combined parameter to return the “best” value. This framework is abstract enough that various problems can be interpreted as “function optimization” problems [2]. However, traditional function optimization is only used to solve some small-scale problems, which are often not applicable in practice. Therefore, people put their eyes on nature, which provides rich models to solve these problems (such as fireflies, bats, and ants). By simulating the natural biological systems, many swarm intelligent optimization algorithms are proposed to solve the application problems with nontraditional methods [3]. Many swarm intelligent optimization algorithms have been proposed, such as particle swarm optimization (PSO) algorithm [4], ant colony optimization (ACO) algorithm [5], bat algorithm (BA) [6], social learning optimization (SLO) algorithm [7], chicken swarm optimization (CSO) algorithm [8], firefly algorithm (FA) [9], and so on.

The bat algorithm (BA) is a heuristic search algorithm proposed by Professor Yang in 2010 based on swarm intelligence [10]. The BA algorithm has many advantages, such as simplicity, fewer parameters, robustness, ease of implementation, and so on. Therefore, due to its obvious superiority, BA has been applied in various application fields, such as the optimal independent micro-smart grid, multiobjective function optimization based on artificial neural network model, economic scheduling problem, economic load scheduling for wind power generation system, fault diagnosis on low-speed rolling bearing, and the optimization of echo state network [11–15]. However, some research reports show that with the increase of problem dimensions, its performance may decline and its exploration capacity may become poor, so it is almost impossible to converge to the global optimal solution. In order to overcome this shortcoming, many scholars have studied and applied this algorithm and proposed corresponding improvement strategies.

An elite crossover binary bat algorithm proposed in [16], the algorithm using the cross mechanism in the elite strategy and genetic algorithm, according to the certain proportion selection in the bat group of elite individuals to crossover, will be a child of the merit of bats group and the parent group of mixed, guarantee the diversity of bats and good sex, and improve the global search ability. Zhou et al. [17] applied the cloud model to bat algorithm. Starting from the living and predation characteristics of bats, the echolocation model is reconstructed, and the transformation theory of cloud model is used to describe the qualitative concept: bats are close to prey. Fister et al. [18] developed the hybrid self-adaptive bat algorithm (HSABA) on the basis of self-adaptive bat algorithm (SABA). The HSABA was combined with the local search heuristic algorithm. The hybrid algorithm was crossbred with different DE strategies and used as the local search heuristic algorithm to improve the current optimal solution, and the solution group was pointed to a better area in the search space. Qu et al. [19] integrated the weed growth, reproduction, spatial diffusion, and competition mechanism of the invasive weed algorithm into the bat algorithm and dynamically adjusts the standard deviation of the weed spatial diffusion operator so that the algorithm not only increases the global search ability but also improves the local search ability. Wu et al. [20] proposed an elite crossover binary bat algorithm. Based on the crossover mechanism of elite strategy and genetic algorithm, this algorithm selects elite individuals in the bat group to cross over according to a certain proportion and then makes a mixed selection between the daughter bat group and parent bat group so as to ensure the diversity and excellence of the bat group and improve the global search ability.

In this paper, a multipopulation parallel bat algorithm based on island model is proposed and applied to three strategies including chaos [21], Levy flight search [22], and contraction factor. Simulation results show that the proposed algorithm is more effective than single chaos, Levy flight search, and contraction factor in terms of convergence speed and accuracy.

## 2. Bat Algorithm

### 2.1. Basic Principle of Bat Algorithm

The bat algorithm (BA) is a new swarm intelligence optimization algorithm, which simulates the foraging behavior of bats. Its principle is to use the bat's advanced echolocation capability [10]. Echolocation is a kind of sonar: the bat (the main small bat) emits a loud and short pulse sound. When the sound hits an object, the echo will return to their ears in a short period of time; bats receive and detect the position of the prey in this way. In order to simulate the foraging process of bats, the biological mechanism of the bat algorithm is described as follows. All bats adopt echolocation to detect distances, and the method used to identify obstacles and prey is difficult to understand. Based on the variable length waves *λ*, loudness *A*_0_, and fixed frequency *f*_min_, the bat searches for the prey with the velocity *V*_*i*_ at the position *X*_*i*_. The bat adjusts the pulse wavelength according to the distance between itself and the prey. On the other hand, when it is close to the prey, the frequency of the transmission *r* ∈ (0,1) will also be adjusted. The loudness changes from the maximum value *A*_0_ to the minimum value *A*_min_ in the searching process.

BA's development takes advantage of existing algorithms and other interesting features, inspired by the wonderful behavior of miniature bat echolocation. Based on these assumptions, this algorithm generates a set of solutions in a random manner and then uses the loop search to find the optimal solution. During this period, the local search is adopted. That is to say that around the optimal solution, the local solution is generated by random flight and produces a global optimal solution. For bats, if their foraging space belongs to the *d*-dimension at the *t* − 1 moment, the position of the bat *i* is *X*_*i*_^*t*−1^, the flight velocity is *V*_*i*_^*t*−1^, and the current global optimal position is *X*^*∗*^. So, the position and flight velocity of bat *i* at time *t* can be updated by

(1) $$\begin{array}{c} f_{i}=f_{\min}+\left(f_{\max}-f_{\min}\right)\beta , \end{array}$$

(2) $$\begin{array}{c} V_{i}^{t}=V_{i}^{t-1}+\left(X_{i}^{t-1}-X^{*}\right)f_{i}, \end{array}$$

(3) $$\begin{array}{c} X_{i}^{t}=X_{i}^{t-1}+V_{i}^{t}, \end{array}$$

where the minimum frequency of the sound waves generated by the bat is *f*_min_ and the maximum frequency is *f*_max_. *β* is a uniformly distributed random number located in the scope [0, 1].

In the initial setting process, the frequency of the bat's emitted sound waves is uniformly distributed in [*f*_min_, *f*_max_]. The corresponding frequency is obtained according to equation (1), and then the local search is carried out according to equations (2) and (3). The bat randomly walks according to the optimal solution, and the new solution is generated by the following equation:

(4) $$\begin{array}{c} X_{\text{new}}=X_{\text{old}}+\varepsilon \bar{A}\left(t\right), \end{array}$$

where *ε* is a random number located in [−1, 1], *X*_old_ represents the solution selected from the current optimal solutions in a random manner, and $\bar{A}\left(t\right)$ refers to the average loudness that the bat produces when the number of iterations is *t*.

By analyzing the loudness *A*_*i*_ and rate *r*_*i*_ of the bat pulse emission, it is found that the update rule can be described as follows. If the bat is aware of the presence of the prey, it will reduce the response of its pulsed emission and increase its pulse emission rate. The loudness *A*_*i*_ and rate *r*_*i*_ of the bat launch pulse are updated by the following equations:

(5) $$\begin{array}{c} r_{i}^{A_{i}^{t+1}=\alpha A_{i}^{t}}, \end{array}$$

(6) $$\begin{array}{c} r_{i}^{t+1}=r_{i}^{t}\left[1+\exp\;yt\right], \end{array}$$

where *r*_*i*_^0^ is the initial rate and *A*_*i*_^0^ is the initial loudness, which are all randomly chosen. *α* and *y* are constants (0 < *α* < 1, *y* > 0).

### 2.2. Pseudocode of the Bat Algorithm

The pseudocode of the bat algorithm is described as follows [18]:

  Input: Bat population *x*_*i*_=(*x*_*i*1_,…,*x*_*i* *D*_)^T^ for *i*=1,…, Np, MAX_FE.
  Output: The best population *x*_best_ and its corresponding value *f*_min_=min(*f*(*x*)).
   init_bat ();
   eval = evaluate the new population;
   *f*_min_ = find_best_solution (*x*_best_); {initialization}
   while termination_condition_not_meet do
    for *i*=1 to Np do
     *y* = generate_new_solution (*x*_*i*_);
    if rand (0, 1) > *r* then
     *y* = improve_the_best_solution (*x*_*i*_);
    end if {local search}
    if *f*_new_ = evaluate_new_solution (*y*);
    eval = eval + 1;
    if *f*_new_ ≤ *f*_*i*_ and *N* (0, 1) < *A* then
     *x*_*i*_=*y*; *f*_*i*_=*f*_new_
    end if {simulated annealing}
    *f*_min_ = find the best solution (*x*_best_);
    end for
   end while

## 3. Improved Bat Algorithm

### 3.1. Bat Algorithm Based on Chaotic Mapping

The chaotic motion is a highly unstable motion in a deterministic system that is limited to a finite phase space. Chaos is a form of aperiodic motion, which is unique and extensive in nonlinear systems [23]. Chaotic systems are very common in natural systems and social systems, which has a complex, random, and accurate characteristic [24]. By analyzing the logistic equation, it is known as the most typical of chaotic systems:

(7) $$\begin{array}{c} S_{k+1}=\mu S_{k}\left(1-S_{k}\right), \end{array}$$

where *μ* is a constant and *S* ∈ (0,1). So, the determined sequence  *S*_0_, *S*_1_,…, can be obtained, and the system is a chaotic system.

There are 10 typical kinds of chaotic mapping [25]. An optimal chaotic mapping method is selected by carrying out the simulation experiments on the test functions. In order to verify the performance of various improved algorithms, a total of seven functions were selected for simulation experiments. The name, expression, and domain range of these functions are shown in Table 1.

**Figure 1:**
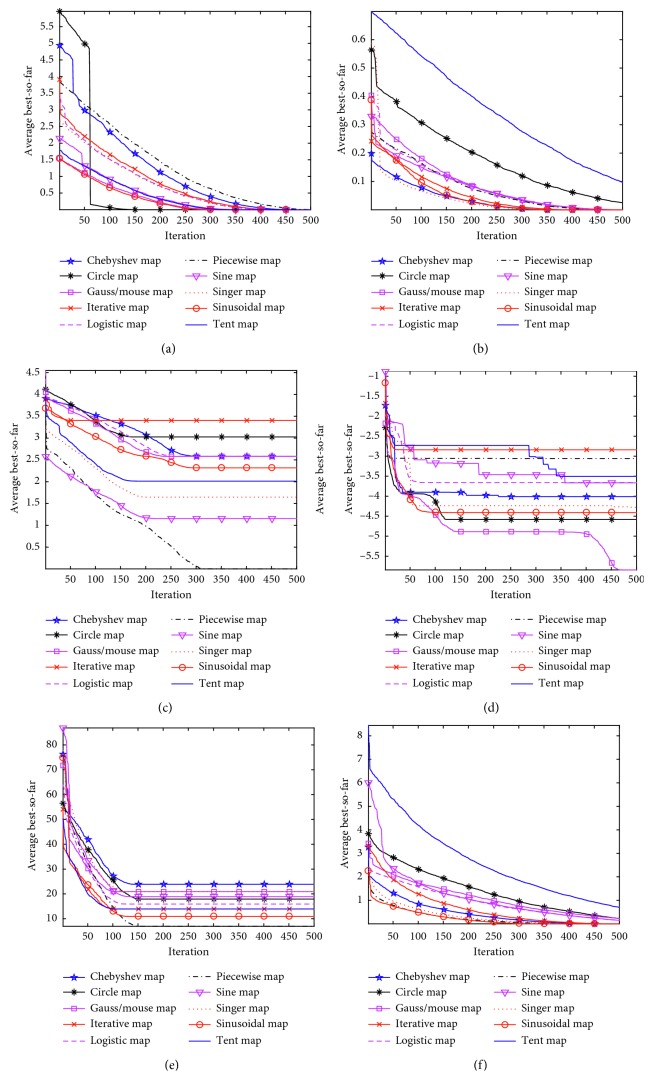
Convergence curves under ten chaotic mappings. (a) *F*_1_. (b) *F*_2_. (c) *F*_3_. (d) *F*_4_. (e) *F*_5_. (f) *F*_6_.

In this section, the first six functions are adopted to carry out the simulation experiments. The simulation results of BA based on the different chaotic mappings is shown in Figure 1, and the performance comparison results are listed in Table 2. By considering the fluctuating feature of the chaotic mapping and the influence of the initial value, the initial point in all chaotic mappings is set as 0.7.

It can be seen from the simulation results on six function optimization problems that the convergence speed and optimization ability of the piecewise chaotic mapping are the best, the optimal value can be found, and the fluctuation is relatively small. Although the optimal value is not obtained in function *F*_6_, the overall trend deviation is not large. So, a chaotic bat algorithm (CBA) is proposed based on the piecewise chaotic mapping shown in Table 3.

It can be seen from the simulation results on six function optimization problems that the convergence speed and optimization ability of the piecewise chaotic mapping are the best, the optimal value can be found, and the fluctuation is relatively small. Although the optimal value is not obtained in functions *F*_1_ and *F*_6_, the overall trend deviation is not large. So, a chaotic bat algorithm (CBA) is proposed based on the piecewise chaotic mapping shown in Table 3.

### 3.2. Bat Algorithm Based on Levy Flight Search Strategy

The study found that Levy's flight behavior in nature is based on the ideal way for food seekers to find food in an unfocused and unpredictable environment, which includes short-range exploratory bounce and occasional long walks. Viswanathan et al. [26] studied the foraging behavior of the albatross and found the same flight path as the Levy flight. Reynolds et al. [27, 28] observed the foraging trajectory of bees and fruit flies and found that the flight trajectory also shows the characteristics of Levy's flight, and even human behavior is similar to the existence of Levy's flight behavior. A series of studies have confirmed that Levy's flight behavior is the best searching strategy for *N* independent explorers when the target position is in a random state and the distribution is relatively loose. A Levy flight-based bat algorithm (LBA) is proposed in this paper.

**Figure 2:**
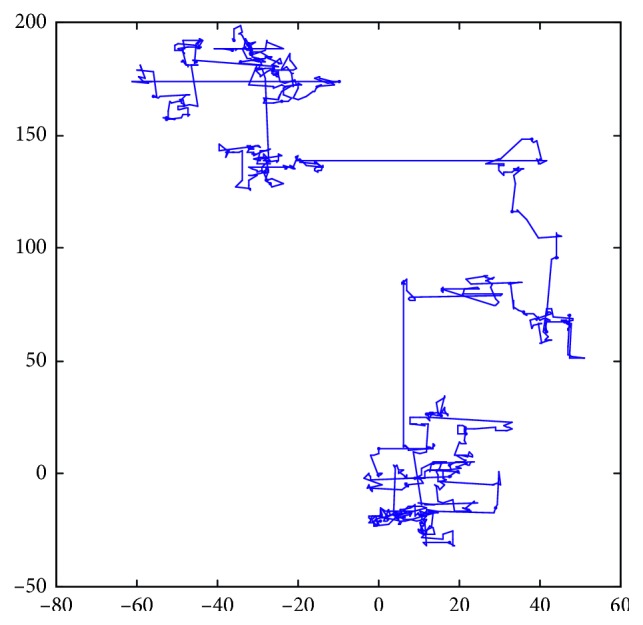
Levy flight tracks.

From a mathematical point of view, Levy flight behavior reflects a class of non-Gaussian stochastic processes, whose steady increments obey the stable distribution of Levy and whose flight path simulation is shown in Figure 2. It can be seen from Figure 2 that it can jump a lot in Levy's searching process and change direction many times, so it can make an individual bat effectively avoid being bound by local attractions and expand the searching space.

Combined with the bat's echolocation feature, it helps to significantly and effectively improve the performance of the bat algorithm. Therefore, the improved algorithm replaces equation (3) with the following equation:

(8) $$\begin{array}{c} X_{i}^{t}=X_{i\;}^{t-1}+l\overset{˙}{e}\text{vy }X\left(X_{i}^{t-1}-X^{*}\right)+V_{i}^{t}. \end{array}$$

The Levy flight is used to replace the local searching for the optimal position of an individual bat, which generates a larger matching and optimization iterations in the global search process so as to make the situation of falling into local optimum improve and also make the convergence accuracy of the algorithm improve.

### 3.3. Bat Algorithm Based on Shrink Factor

For the bat algorithm, since the individual is flying toward the optimal solution during optimization, there is usually a problem of early convergence of the bats, so it is difficult to obtain better optimization results. In order to avoid the premature convergence problem of bat algorithm and make the individual converge to the global optimal solution quickly, a contraction factor is proposed to realize the shrink factor bat algorithm (SBA), which not only maintains the diversity of the population but also improves the convergence efficiency. The proposed SBA can be realized by the following equations:

(9) $$\begin{array}{c} k=\frac{2}{\left|2-d-\sqrt{d^{2}-4d}\right|}, \end{array}$$

(10) $$\begin{array}{c} V_{i}^{t}=V_{i}^{t-1}+\left(X_{i}^{t-1}-X_{*}\right)f_{i}\cdot {}^{*}k, \end{array}$$

where *d* is a constant, and the value of the *d* in this experiment is 10.

### 3.4. Multipopulation Parallel Bat Algorithm Based on Island Model

The algorithm based on the parallel model has the following two characteristics. The first is to break down a group into multiple groups by realizing a divide-and-conquer approach. The second is to control and manage the information exchange among the subgroups. From the perspective of parallel algorithms, this structural difference produces three parallel population models: master-slave parallel model, island model, and adjacency model [29]. The adjacency model and the island model belong to the decomposition parallel scheme, which divides the entire group into several subgroups. Each subgroup is distributed on its own processor for subgroup evolution, and each processor exchanges information at the appropriate time. The adjacency model is also known as a fine-grained model, with only one individual in each subgroup. The island model, also known as the coarse-grained model, has multiple subgroups on each processor. The coarse-grained models are easy to implement and can be simulated on networks or stand-alone systems without parallel computers, so coarse-grained models are the most commonly used in parallel algorithms [30].

**Figure 3:**
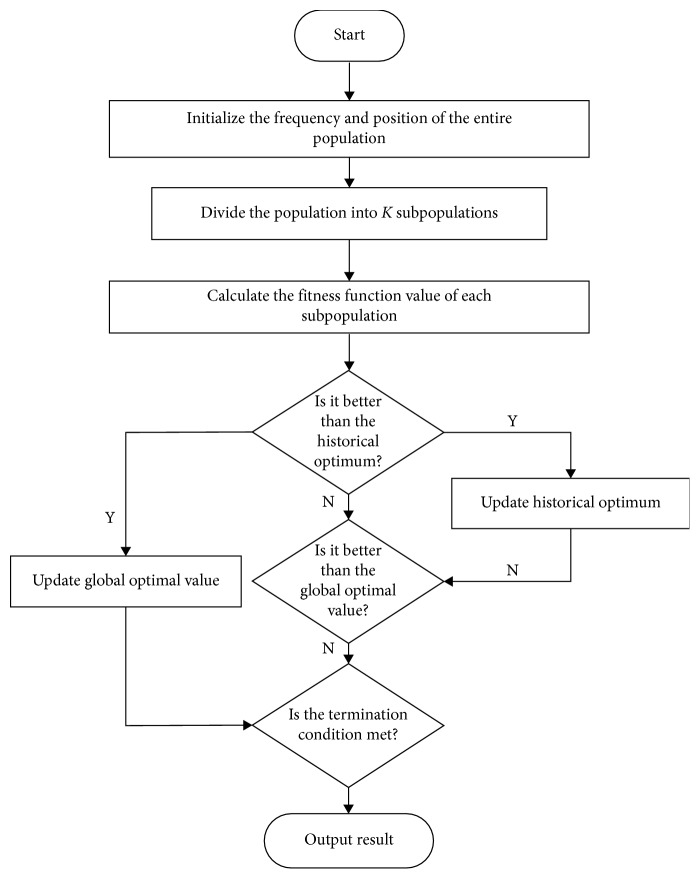
Bat algorithm flow chart based on island model.

In this paper, an island multipopulation parallel bat algorithm (IBA) is proposed by adopting the parallel optimization scheme and introducing a centralized information migration strategy. The entire population is divided into many subgroups. Each subgroup performs a global search only on the island, and the suitability of each individual in the island is calculated and evaluated to produce the best individual in the island. The entire evolution of the island is realized by using a separate subprocess to reduce the degree of coupling. Each subprocess uses a centralized migration strategy to periodically send the best individual in the island to the main process to form the main process, and the main process selects the global best individual to from the entire population so as to broadcast to the subprocess, which will force the subpopulation to perform the global most excellent evolution. The flow chart of the algorithm is shown in Figure 3.

## 4. Simulation Experiment and Result Analysis

The multipopulation idea based on the island model is integrated into the other three improved bat algorithms (SBA, LBA, and CBA) to form the island multipopulation chaotic bat algorithm (CBAS), the island multipopulation Levy flight bat algorithm (LBAS), and the island multipopulation shrink factor bat algorithm (SBAS). The seven algorithms (IBA, SBA, LBA, CBA, CBAS, LBAS, and SBAS) were adopted to carry out the simulation experiments on twelve typical test functions (*F*_1_, *F*_2_, *F*_3_, *F*_5_, *F*_6_, *F*_7_, *F*_8_, *F*_9_, *F*_10_, and *F*_11_) shown in Table 1. The performance of the algorithms was evaluated by counting the optimal value, the average values, and the convergence curves of the corresponding functions in 10 runs. The parameter settings of the algorithm are shown in Table 4.

**Figure 4:**
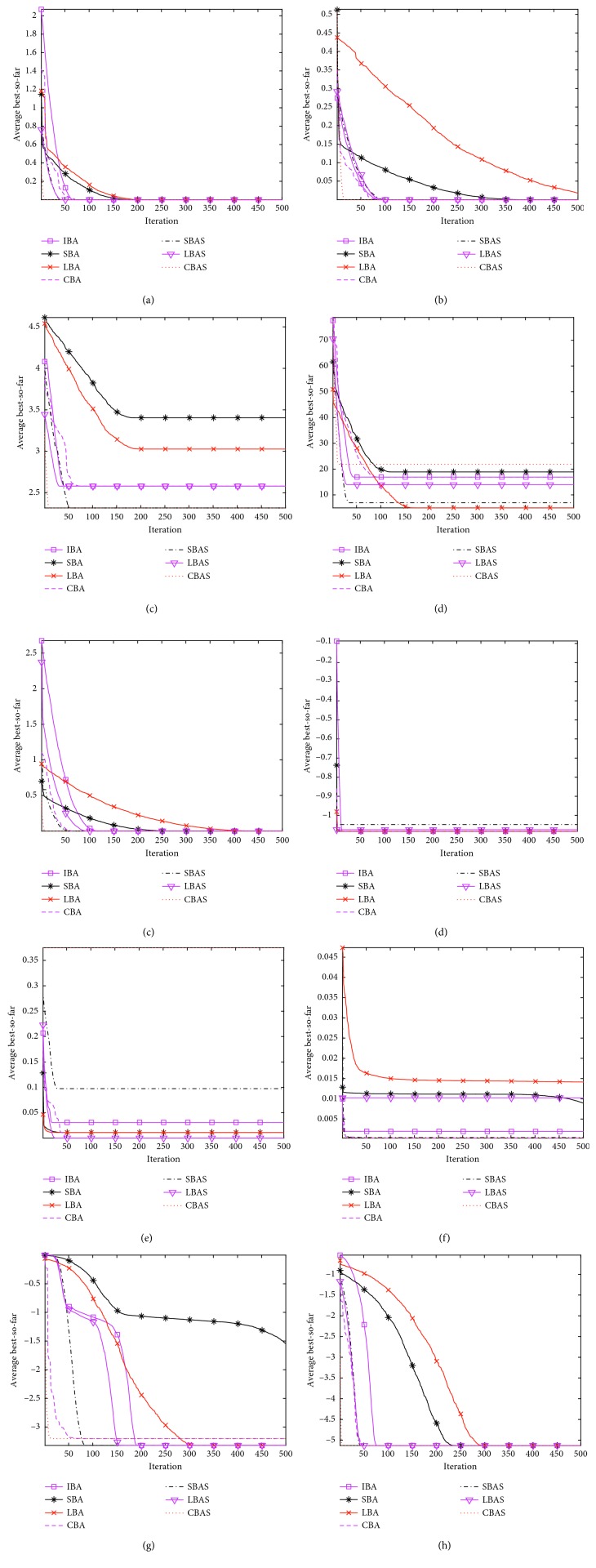
Convergence curves for typical function optimization problems under seven algorithms. (a) *F*_1_. (b) *F*_2_. (c) *F*_3_. (d) *F*_5_. (e) *F*_6_. (f) *F*_7_. (g) *F*_8_. (h) *F*_9_. (i) *F*_10_. (j) *F*_11_.

The performance comparison results are listed in Table 5, and the function convergence curves are shown in Figure 4.

It can be seen from the simulation results that the overall searching ability of IBA, SBAS, LBAS, and CBAS is better than that of the original algorithms (BA, SBA, LBA, and CBA), which have less volatility and relatively stable performance. From the perspective of a single function, most algorithms corresponding to each function are different, which proves that the seven different algorithms have different optimization capabilities for the same problem. SBAS obtains the optimal value for test functions *F*_1_, *F*_6_, and *F*_7_. IBA obtains the optimal value for test function *F*_3_. LBA obtains the optimal value for functions *F*_5_ and *F*_2_. All seven optimization algorithms get the global optimal value for test function *F*_3_. It can be seen from the comparison of the convergence curves of the six test functions that overall, the convergence speed of the multipopulation algorithms (IBA, SBAS, and LBAS) is faster than that of the original algorithms. However, the multipopulation algorithm CBAS is slower than the original algorithms. For the test functions *F*_1_ and *F*_2_, SBAS converges faster. For test functions *F*_6_ and *F*_7_, LBAS converges faster. For function *F*_3_, CBAS converges faster. For function *F*_5_, IBA algorithm converges faster. The convergence rate of the function is relatively larger at an early stage, and there is a delay phenomenon in the later stage although the speed is slowed down.

It can be seen from the simulation results that the overall searching ability of IBA, SBAS, LBAS, and CBAS is better than that of the original algorithms (BA, SBA, LBA, and CBA), which have less volatility and relatively stable performance. From the perspective of a single function, most algorithms corresponding to each function are different, which proves that the seven different algorithms have different optimization capabilities for the same problem. SBAS obtains the optimal value for test functions *F*_2_, *F*_5_, *F*_9_, *F*_12_, and *F*_13_. SBA obtains the optimal value for test functions *F*_3_ and *F*_8_. LBAS obtains the optimal value for test functions *F*_1_, *F*_7_, and *F*_12_. It can be seen from the comparison of the convergence curves of the ten test functions that overall, the convergence speed of the multi-population algorithms (IBA, SBAS, and CBAS) is faster than that of the original algorithms. For the test functions *F*_5_ and *F*_10_, SBAS converges faster. For the test functions *F*_8_ , LBAS converges faster. For the test functions *F*_1_, *F*_2_, *F*_3_, *F*_6_, *F*_9_, and *F*_11_, CBAS converges faster. The convergence rate of the function is relatively larger at an early stage, and there is a delay phenomenon in the later stage although the speed is slowed down.

## 5. Conclusions

Based on the concept of chaotic mapping, the optimal chaotic mapping is selected to produce the chaotic bat algorithm. The LBA algorithm is proposed by adopting the Levy flight searching strategy. The shrink factor is proposed to realize SBA algorithm, and the multipopulation parallel bat algorithm (IBA) based on island is proposed. The performance is compared by using seven test functions under seven optimization algorithms (IBA, SBA, LBA, CBA, SBAS, LBAS, and CBAS). The comparison results show that the multipopulation algorithms (IBA, SBAS, LBAS, and CBAS) are better than the original algorithms and have relatively stable performance in both the optimization ability and the convergence speed. The experiment proves that the improved algorithm has better optimization precision and convergence speed, which can make up for the deficiency of the original algorithm.

## Figures and Tables

**Table 1:** Simulation test functions.

Function	Expression	Range	Minimum value
*F*_1_	*f*_1_(*x*)=∑_*i*=1_^*d*^*x*_*i*_^2^	[−100, 100]	0
*F*_2_	$f_{2}\left(x\right)=\sum_{i=1}^{n}\left(x_{i}^{2}/4000\right)-\prod_{i=1}^{n}\cos\left(x_{i}/\sqrt{i}\right)+1$	[−600, 600]	0
*F*_3_	$f_{3}\left(x\right)=20+e-20e^{\left(-0.2\sqrt{1/n}\sum_{i=1}^{n}x_{i}^{2}\right)}-e^{\left(\left(1/n\right)\sum_{i=1}^{n}\cos\left(cx_{i}\right)\right)}$	[−32, 32]	0
*F*_4_	*f*_4_(*x*)=−∑_*i*=1_^*d*^sin(*x*_*i*_)[sin(*ix*_*i*_^2^/*π*)]^2*m*^, *m*=10	[0, *π*]	0
*F*_5_	*f*_5_(*x*)=∑_*i*=1_^*n*^[*x*_*i*_^2^ − 10 cos(2*πx*_*i*_)+10]	[−5.12, 5.12]	
*F*_6_	*f*_6_(*x*)=∑_*i*=1_^*d*^∑_*j*=1_^*i*^*x*_*j*_^2^	[−65.536, 65.536]	0
*F*_7_	*f*_7_(*x*)=(*x*_1_ − 1)^2^+∑_*i*=2_^*d*^*i*(2*x*_*i*_^2^ − *x*_*i*−1_)^2^	[−5 10]	0
*F*_8_	$\begin{array}{c} f_{8}\left(x\right)=0.1\left\{\sin^{2}\left(3\pi x_{1}\right)+\sum_{i=1}^{n}{\left(x_{i}-1\right)}^{2}\left[1+\;\sin^{2}\left(3\pi x_{i}+1\right)\right]+{\left(x_{n}-1\right)}^{2}\left[1+\;\sin^{2}\left(2\pi x_{n}\right)\right]\right\}+\sum_{i=1}^{n}u\left(x_{i},5,100,4\right)\\ u\left(x_{i},a,k,m\right)=\left\{\begin{array}{cc} k{\left(x_{i}-a\right)}^{m} & x_{i}>a\\ 0 & -a<x_{i}<a\\ k{\left(-x_{i}-a\right)}^{m} & x_{i}<-a \end{array}\right. \end{array}$	[−50, 50]	0
*F*_9_	$f_{9}\left(x\right)={\sum_{i=1}^{11}\left[a_{i}-\frac{x_{1}\left(b_{i}^{2}+b_{i}x_{2}\right)}{b_{i}^{2}+b_{i}x_{3}+x_{4}}\right]}^{2}$	[−5, 5]	0.00030
*F*_10_	*f*_10_(*x*)=−∑_*i*=1_^4^*c*_*i*_exp(−∑_*j*=1_^6^*a*_*ij*_(*x*_*j*_ − *p*_*ij*_)^2^)	[0, 1]	−3.32
*F*_11_	*f*_11_(*x*)=−∑_*i*=1_^10^[(*X* − *a*_*i*_)(*X* − *a*_*i*_)^T^+*c*_*i*_]^−1^	[0, 10]	−10.536

**Table 2:** Simulation results on function optimization problems under different chaotic mappings.

Function	Chaotic mapping	Avg.	Best	Std.
*F*_1_	Chebyshev map	2.46*E* − 03	6.07*E* − 07	7.37*E* − 03
	Circle map	4.08*E* − 03	9.77*E* − 07	1.12*E* − 02
	Gauss/mouse map	1.77*E* − 04	6.33*E* − 07	5.25*E* − 04
	Iterative map	1.92*E* − 02	1.22*E* − 06	5.76*E* − 02
	Logistic map	1.74*E* − 06	1.10*E* − 06	5.28*E* − 07
	Piecewise map	1.91*E* − 06	5.70*E* − 07	7.10*E* − 07
	Sine map	1.60*E* − 02	9.53*E* − 07	4.76*E* − 02
	Singer map	3.38*E* − 02	1.14*E* − 06	1.01*E* − 01
	Sinusoidal map	8.43*E* − 03	1.07*E* − 06	2.53*E* − 02
	Tent map	**2.25E** **−** **06**	1.21*E* − 06	7.34*E* − 07

*F*_2_	Chebyshev map	1.22*E* − 03	1.67*E* − 07	3.01*E* − 03
	Circle map	2.06*E* − 03	1.27*E* − 07	5.76*E* − 03
	Gauss/mouse map	6.44*E* − 03	1.28*E* − 07	1.47*E* − 02
	Iterative map	3.04*E* − 03	1.28*E* − 07	6.60*E* − 03
	Logistic map	7.70*E* − 05	8.32*E* − 08	1.66*E* − 04
	Piecewise map	**2.28E** **−** **06**	1.33*E* − 07	6.04*E* − 06
	Sine map	3.75*E* − 03	4.52*E* − 08	1.12*E* − 02
	Singer map	8.91*E* − 03	1.16*E* − 07	1.33*E* − 02
	Sinusoidal map	4.62*E* − 03	2.06*E* − 07	9.24*E* − 03
	Tent map	9.79*E* − 04	8.08*E* − 08	2.58*E* − 03

*F*_3_	Chebyshev map	2.11*E* + 00	1.16*E* + 00	5.77*E* − 01
	Circle map	2.13*E* + 00	1.16*E* + 00	9.04*E* − 01
	Gauss/mouse map	2.01*E* + 00	1.42*E* − 03	7.21*E* − 01
	Iterative map	2.24*E* + 00	1.16*E* + 00	7.99*E* − 01
	Logistic map	2.23*E* + 00	1.91*E* − 03	9.84*E* − 01
	Piecewise map	**1.89E** **+** **00**	1.59*E* − 03	6.80*E* − 01
	Sine map	2.41*E* + 00	1.16*E* + 00	6.13*E* − 01
	Singer map	1.95*E* + 00	1.64*E* − 03	7.85*E* − 01
	Sinusoidal map	2.22*E* + 00	1.76*E* − 03	9.29*E* − 01
	Tent map	1.90*E* + 00	1.31*E* − 03	7.28*E* − 01

*F*_4_	Chebyshev map	−4.3381	−3.4831	0.4727
	Circle map	−4.2815	−2.8800	0.9298
	Gauss/mouse map	−4.4270	−3.3986	0.7517
	Iterative map	−4.3976	−3.2347	0.7557
	Logistic map	−4.6527	−3.5362	0.9810
	Piecewise map	**−3.6912**	−3.3934	0.1789
	Sine map	−4.1871	−3.3551	0.5061
	Singer map	−3.9540	−3.2770	0.5729
	Sinusoidal map	−4.1621	−2.6385	0.7561
	Tent map	−4.0751	−2.6231	0.9906

*F*_5_	Chebyshev map	12.3377	6.9650	4.9388
	Circle map	14.6261	6.9650	5.0547
	Gauss/mouse map	13.4322	5.9700	5.1170
	Iterative map	11.6412	4.9750	4.6038
	Logistic map	12.0392	3.9801	4.3472
	Piecewise map	**9.6514**	6.9649	1.6074
	Sine map	13.1337	4.9750	5.5895
	Singer map	14.0292	7.9600	3.9410
	Sinusoidal map	13.0342	6.9649	6.4242
	Tent map	13.5317	6.9650	5.2686

*F*_6_	Chebyshev map	2.64*E* − 01	3.69*E* − 06	2.98*E* − 01
	Circle map	5.17*E* − 01	1.08*E* − 05	8.15*E* − 01
	Gauss/mouse map	1.84*E* − 01	4.49*E* − 05	1.45*E* − 01
	Iterative map	1.92*E* − 01	1.64*E* − 05	1.69*E* − 01
	Logistic map	3.22*E* − 01	2.76*E* − 03	3.84*E* − 01
	Piecewise map	1.49*E* − 01	6.80*E* − 06	1.90*E* − 01
	Sine map	1.75*E* − 01	2.25*E* − 06	2.58*E* − 01
	Singer map	2.17*E* − 01	2.46*E* − 06	2.55*E* − 01
	Sinusoidal map	**1.19E** **−** **01**	2.12*E* − 06	1.72*E* − 01
	Tent map	2.02*E* − 01	7.22*E* − 06	2.21*E* − 01

**Table 3:** Expression of piecewise map.

Chaotic mapping	Expression	Range
Piecewise map	$x_{i+1}=\left\{\begin{array}{c} x_{i}/P\;\left[0,P\right)\\ x_{i}-P/0.5-P\;\left[P,0.5\right)\\ 1-P-x_{i}/0.5-P\;\left[0.5,1-P\right)\\ 1-x_{i}/P\;\left[1-P,1\right)\\ P=0.4 \end{array}\right.$	(0, 1)

**Table 4:** Parameter settings of the algorithm.

Name of parameter	Parameter values
Population size	*n*=30
Maximum number of iterations	Max *T*=500
Loudness	*A*=0.5
Rate	*r*=0.5
Maximum frequency	*f*_max_=2
Minimum frequency	*f*_min_=0

**Table 5:** Performance comparison results under seven algorithms.

Function	Optimization method	Optimal solution	Average	Standard deviation
*F*_1_	IBA	3.4364*E* − 08	3.3842*E* − 01	6.7955*E* − 01
	SBA	3.0580*E* − 09	3.0762*E* − 08	3.0405*E* − 08
	LBA	4.4310*E* − 08	1.0789*E* − 07	5.6368*E* − 08
	CBA	2.9439*E* − 20	1.3670*E* − 15	3.1453*E* − 15
	SBAS	4.2826*E* − 09	9.0180*E* − 03	2.7054*E* − 02
	LBAS	9.1300*E* − 09	**4.8765E** **−** **08**	2.9546*E* − 08
	CBAS	3.3146*E* − 09	2.0426*E* − 08	1.4284*E* − 08

*F*_2_	IBA	6.6230*E* − 08	5.9714*E* − 02	1.7914*E* − 01
	SBA	7.3645*E* − 08	8.5002*E* − 03	1.2582*E* − 02
	LBA	2.1114*E* − 07	9.9413*E* − 03	2.4116*E* − 02
	CBA	2.2553*E* − 08	1.9696*E* − 08	1.3041*E* − 08
	SBAS	3.3806*E* − 08	**6.1948E** **−** **08**	1.8490*E* − 08
	LBAS	6.3657*E* − 08	1.0919*E* − 07	2.4972*E* − 08
	CBAS	1.8559*E* − 08	6.6582*E* − 02	1.9975*E* − 01

*F*_3_	IBA	2.3169	2.9481	0.4250
	SBA	0.0009	**1.9313**	0.8860
	LBA	1.1552	2.1514	0.6486
	CBA	1.1551	2.3774	0.5599
	SBAS	2.0133	2.3912	0.3715
	LBAS	2.3169	2.8544	0.4626
	CBAS	1.6462	2.7592	0.7613

*F*_5_	IBA	5.9699	15.5719	13.7849
	SBA	3.9899	9.9497	5.6459
	LBA	7.9598	13.4321	3.3074
	CBA	3.9798	12.8350	6.8421
	SBAS	**3.9800**	7.2633	2.3566
	LBAS	5.9699	8.5568	1.7909
	CBAS	4.9748	19.5561	16.6536

*F*_6_	IBA	3.8782*E* − 08	4.4456*E* − 01	9.5610*E* − 01
	SBA	5.8307*E* − 09	1.1123*E* − 04	2.9158*E* − 04
	LBA	8.6362*E* − 08	1.1877*E* − 02	2.3393*E* − 02
	CBA	1.0664*E* − 19	**3.4219E** **−** **17**	6.0237*E* − 17
	SBAS	1.3461*E* − 08	5.0505*E* − 08	2.2872*E* − 08
	LBAS	2.8347*E* − 08	1.7624*E* − 01	5.2873*E* − 01
	CBAS	3.2492*E* − 09	2.5676*E* − 08	1.5720*E* − 08

*F*_7_	IBA	−0.0916	−0.8837	0.3961
	SBA	−1.0833	−1.0833	0.0000
	LBA	−1.0833	−1.0833	0.0000
	CBA	−1.0833	−1.0833	0.0000
	SBAS	−0.6009	−1.0315	0.2153
	LBAS	1.0416	**−0.7575**	0.8996
	CBAS	0.2604	−0.8475	0.5539

*F*_8_	IBA	3.8153*E* − 09	1.3534*E* − 01	1.2480*E* − 01
	SBA	1.8240*E* − 10	**2.9037E** **−** **02**	5.6482*E* − 02
	LBA	1.3140*E* − 08	2.0437*E* − 02	2.1852*E* − 02
	CBA	2.1564*E* − 31	1.9632*E* − 02	2.5663*E* − 02
	SBAS	1.2837*E* − 10	2.4524*E* − 02	2.0517*E* − 02
	LBAS	1.4992*E* − 09	1.3792*E* − 01	1.4911*E* − 01
	CBAS	1.3498*E* − 32	1.3242*E* − 01	1.9408*E* − 01

*F*_9_	IBA	0.0003	0.0090	0.0159
	SBA	0.0003	0.0016	0.0026
	LBA	0.0003	0.0033	0.0073
	CBA	0.0003	0.0006	0.0003
	SBAS	0.0003	**0.0003**	5.6425*E* − 05
	LBAS	0.0005	0.0028	0.0029
	CBAS	0.0003	0.0007	0.0005

*F*_10_	IBA	−3.3220	−3.0019	0.9602
	SBA	−3.3220	−2.8942	0.6939
	LBA	−3.3220	−3.0492	0.7143
	CBA	−3.2031	−3.2507	0.0582
	SBAS	−3.2031	**−3.2863**	0.0545
	LBAS	−3.2031	**−3.2863**	0.0545
	CBAS	−3.2031	−3.2982	0.0476

*F*_11_	IBA	−5.1285	−4.2011	1.8551
	SBA	−5.1285	**−5.1285**	3.6134*E* − 07
	LBA	−5.1285	−4.8311	0.8920
	CBA	−5.1285	−5.1285	4.5201*E* − 15
	SBAS	−5.1285	**−5.1285**	1.5732*E* − 07
	LBAS	−5.1285	−2.8510	1.9806
	CBAS	−5.1285	**−5.1285**	5.7559*E* − 08

## Data Availability

There are no data available for this paper.

## References

[1] Wang G.-G., Guo L., Gandomi A. H., Hao G.-S., Wang H. (2014). Chaotic krill herd algorithm. *Information Sciences*.

[2] Bell N., Oommen B. J. (2017). A novel abstraction for swarm intelligence: particle field optimization. *Autonomous Agents and Multi-Agent Systems*.

[3] Yang X.-S., Deb S., Fong S., He X., Zhao Y.-X. (2016). From swarm intelligence to metaheuristics: nature-inspired optimization algorithms. *Computer*.

[4] Wang L., Geng H., Liu P. (2015). Particle swarm optimization based dictionary learning for remote sensing big data. *Knowledge-Based Systems*.

[5] Gheraibia Y., Djafri K., Krimou H. (2018). Ant colony algorithm for automotive safety integrity level allocation. *Applied Intelligence*.

[6] Adarsh B. R., Raghunathan T., Jayabarathi T., Yang X.-S. (2016). Economic dispatch using chaotic bat algorithm. *Energy*.

[7] Huang F., Li X., Zhang S., Zhang J. (2018). Harmonious genetic clustering. *IEEE Transactions on Cybernetics*.

[8] Wu D., Xu S., Kong F. (2016). Convergence analysis and improvement of the chicken swarm optimization algorithm. *IEEE Access*.

[9] Olatomiwa L., Mekhilef S., Shamshirband S., Mohammadi K., Petković D., Sudheer C. (2015). A support vector machine-firefly algorithm-based model for global solar radiation prediction. *Solar Energy*.

[10] Yang X.-S. (2010). A new metaheuristic bat-inspired algorithm. *Nature Inspired Cooperative Strategies for Optimization (NICSO 2010)*.

[11] Jaddi N. S., Abdullah S., Hamdan A. R. (2015). Multi-population cooperative bat algorithm-based optimization of artificial neural network model. *Information Sciences*.

[12] Duan Y., Zhao M. (2015). Bat algorithm on economic load dispatch in wind power system. *Electrical Engineering*.

[13] Zhao D., He Y. (2015). Chaotic binary bat algorithm for analog test point selection. *Analog Integrated Circuits and Signal Processing*.

[14] Kang M., Kim J., Kim J.-M. (2015). Reliable fault diagnosis for incipient low-speed bearings using fault feature analysis based on a binary bat algorithm. *Information Sciences*.

[15] Wang H., Yan X. (2015). Optimizing the echo state network with a binary particle swarm optimization algorithm. *Knowledge-Based Systems*.

[16] Xiao H. H., Duan Y. M. (2014). Research and application of improved bat algorithm based on DE algorithm. *Computer Simulation*.

[17] Zhou Y., Xie J., Li L., Ma M. (2014). Cloud model bat algorithm. *Scientific World Journal*.

[18] Fister I., Fong S., Brest J., Fister I. (2014). A novel hybrid self-adaptive bat algorithm. *Scientific World Journal*.

[19] Qu C. W., Fu Y. M., Hou Y. S. (2015). A bat algorithm based on the fusion of invasive weed operators. *Computer Application and Software*.

[20] Wu C. C., He Y. Z., Chen Y. Y., Liu X., Cai X. (2017). Elite crossover binary bat algorithm for 0-1 knapsack problem. *Application Research of Computers*.

[21] Ma Y. D., Wang W. D., Wen Q. (2015). Transmission network planning based on bat algorithm with chaotic search strategy. *Power System Protection and Control*.

[22] Shi H., Wang W. L., Li Y. J. (2015). Bat algorithm based on Levy flight feature and its localization application in WSN. *Journal of Sensing Technology*.

[23] Cai P., Yuan Z. Z. (2017). Hopf bifurcation and chaos control in a new chaotic system via hybrid control strategy. *Chinese Journal of Physics*.

[24] Huang S. J., Gu P. H., Su W. F., Liu X.-Z., Tai T.-Y. Application of flower pollination algorithm for placement of distribution transformers in a low-voltage grid.

[25] Saremi S., Mirjalili S., Lewis A. (2014). Biogeography-based optimisation with chaos. *Neural Computing and Applications*.

[26] Viswanathan G. M., Afanasyev V., Buldyrev S. V., Murphy E. J., Prince P. A., Stanley H. E. (1996). Lévy flight search patterns of wandering albatrosses. *Nature*.

[27] Reynolds A. M., Smith A. D., Reynolds D. R., Carreck N. L., Osborne J. L. (2007). Honeybees perform optimal scale-free searching flights when attempting to locate a food source. *Journal of Experimental Biology*.

[28] Reynolds A. M., Frye M. A. (2007). Free-flight odor tracking in drosophila is consistent with an optimal intermittent scale-free search. *PLoS One*.

[29] Cantú-Paz E., Goldberg D. E. (2000). Efficient parallel genetic algorithms: theory and practice. *Computer Methods in Applied Mechanics and Engineering*.

[30] Alba E., Troya J. M. (2001). Analyzing synchronous and asynchronous parallel distributed genetic algorithms. *Future Generation Computer Systems*.

